# Bifactor analysis of the Hospital Anxiety and Depression Scale (HADS) in individuals with traumatic brain injury

**DOI:** 10.1038/s41598-023-35017-7

**Published:** 2023-05-17

**Authors:** Jai Carmichael, Gershon Spitz, Kate Rachel Gould, Lisa Johnston, Alexia Samiotis, Jennie Ponsford

**Affiliations:** 1 Monash-Epworth Rehabilitation Research Centre, Epworth HealthCare, Melbourne, Australia; 2 School of Psychological Sciences, Turner Institute for Brain and Mental Health, Monash University, Clayton, Australia; 3 Department of Neuroscience, Central Clinical School, Faculty of Medicine, Nursing and Health Sciences, Monash University, Melbourne, Australia

**Keywords:** Brain injuries, Outcomes research, Comorbidities, Disability

## Abstract

Anxiety and depression symptoms are commonly experienced after traumatic brain injury (TBI). However, studies validating measures of anxiety and depression for this population are scarce. Using novel indices derived from symmetrical bifactor modeling, we evaluated whether the Hospital Anxiety and Depression Scale (HADS) reliably differentiated anxiety and depression in 874 adults with moderate-severe TBI. The results showed that there was a dominant general distress factor accounting for 84% of the systematic variance in HADS total scores. The specific anxiety and depression factors accounted for little residual variance in the respective subscale scores (12% and 20%, respectively), and overall, minimal bias was found in using the HADS as a unidimensional measure. Further, in a subsample of 184 participants, the HADS subscales did not clearly discriminate between formal anxiety and depressive disorders diagnosed via clinical interview. Results were consistent when accounting for degree of disability, non-English speaking background, and time post-injury. In conclusion, variance in HADS scores after TBI predominately reflects a single underlying latent variable. Clinicians and researchers should exercise caution in interpreting the individual HADS subscales and instead consider using the total score as a more valid, transdiagnostic measure of general distress in individuals with TBI.

## Introduction

Comorbid anxiety and depression symptoms are common after traumatic brain injury (TBI). Studies have reported that 60–77% of individuals with TBI who have major depressive disorder in the first year post-injury also meet criteria for an anxiety disorder^1–4^. Although comorbid anxiety and depression are also commonly observed in non-TBI populations, especially high rates of comorbidity after TBI may reflect a form of generalized emotional distress in response to a life-altering injury^5^. Additionally, traditional diagnostic-categorical approaches to conceptualizing psychopathology may inflate rates of psychiatric comorbidity^6^. Traditional diagnostic taxonomies, such as the *Diagnostic and Statistical Manual of Mental Disorders* (DSM), organize psychopathological symptoms based on consensus judgments of experts, rather than observed patterns of statistical covariance, leading to substantial symptom overlap between supposedly discrete diagnoses^7–9^. Psychiatric comorbidity presents significant challenges to research designs and clinical decision-making^10^. For example, mental health interventions for TBI are often designed and evaluated based on a single, traditionally defined psychiatric diagnosis, but this does not reflect the complex clinical reality of mental health difficulties faced by this population^11^.

Although alternative frameworks for reliably parsing different components of psychopathology and addressing comorbidity are being developed^8,12^, current mental health assessment tools used for individuals with TBI remain largely tied to traditional diagnostic conceptualizations. Brief self-report measures are more commonly used for assessing anxiety and depression in this population than structured clinical interviews^13,14^. Among these self-report measures, the Hospital Anxiety and Depression Scale (HADS)^15^ is frequently used^14,16^ and recommended by experts for use with individuals with TBI^17^, valued because it is relatively free of physical and cognitive symptoms that are associated with TBI.

Although the HADS was not intended as a diagnostic tool, its developers “aimed to define carefully and distinguish between the concepts of anxiety and depression” (p. 362)^15^. Accordingly, separate subscales for anxiety and depression (seven items each) are scored and interpreted, with clinical cut-offs for each. Despite its intended structure, several prior studies showed that the HADS subscales lacked discriminant validity in individuals with TBI with respect to ‘gold-standard’ DSM-IV anxiety and depressive disorders diagnosed via semi-structured clinical interview^5,18,19^. Moreover, while previous factor analyses found acceptable fit for one-, two-, and three-factor models of the HADS in individuals with TBI, the anxiety and depression factors were strongly positively correlated (*r* = 0.64–0.92)^20–22^. This remained the case even when modeling a higher-order negative affect factor^21^. Further, many items cross-loaded on the anxiety and depression factors in an exploratory factor analysis^22^. These findings raise concerns regarding the ability of the HADS to reliably measure specific factors of anxiety and depression in individuals with TBI. Instead, a total score reflecting general distress may be more justified. This may represent a psychometric limitation of the HADS. A general distress factor of the HADS is also consistent with prior research suggesting the presence of a mixed anxiety/depression construct in neurological samples^23–25^, as well as the emergence of transdiagnostic approaches in the broader fields of psychiatry and clinical psychology^26,27^.

**Figure 1:**
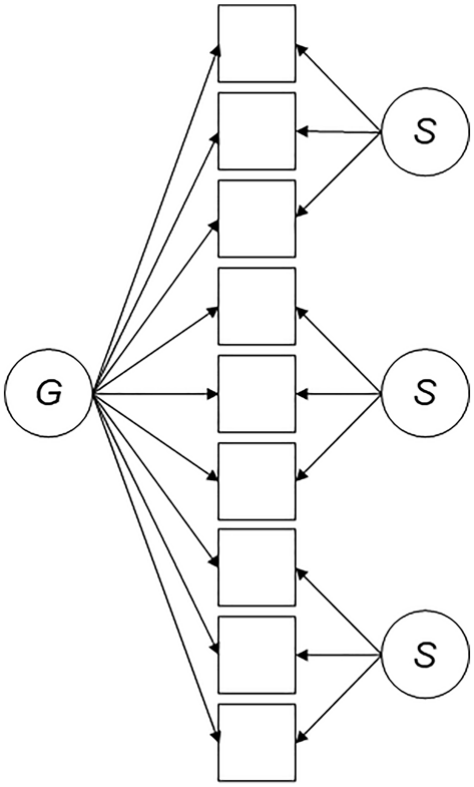
Illustration of a symmetrical bifactor model. The circle labelled ‘G’ is the general factor and the circles labelled ‘S’ are specific factors. Squares represent indicators (e.g., items of an assessment measure). Arrows represent the correlation between a factor and one of its indicators. In a symmetrical bifactor model, every indicator loads on the general factor and on one specific factor. The factors are set to be orthogonal (i.e., uncorrelated), thereby *completely* partitioning the systematic variance into components due to the general and specific factors.

TBI clinicians and researchers typically score and interpret the individual HADS subscales based on first-order correlated factor models which have not accounted for a general factor. However, the validity of these subscales appears tenuous. This study sought to add to our knowledge about assessing psychological distress in individuals with TBI by evaluating the viability of using the HADS subscales in this population more closely. Bifactor analysis is a latent variable approach that can directly answer this research question. In a symmetrical bifactor model (see Fig. 1), each indicator loads on a general factor (e.g., general distress) and on one of several specific factors (e.g., anxiety). All factors are set to be orthogonal (i.e., uncorrelated with one another)^28,29^. Various bifactor statistical indices, such as omega hierarchical reliability coefficients and explained common variance, can be used to quantify the reliability of the general and specific factors and degree of unidimensionality of a psychometric instrument^30^.

Previous research using bifactor statistical indices has found that a general distress factor can account for a large proportion of the systematic variance (72–84%) in HADS total scores in community, medical, and psychiatric populations^31–34^. When holding this general factor constant, the specific anxiety and depression factors accounted for only a small proportion of the residual variance in the corresponding subscale scores (4–19% and 13–48%, respectively). These findings suggest that the HADS total score may be a more valid and conceptually meaningful measure of general distress compared to the subscales for anxiety and depression^24^. However, bifactor statistical indices have not yet been employed to evaluate the HADS in individuals with TBI. In contrast to higher-order factor models, bifactor models *completely* partition systematic variance into components due to a general factor versus specific factors, making them better suited for evaluating the viability of subscale scores compared to a total score^35^.

Using bifactor analysis and associated statistical indices, we aimed to determine whether the HADS can reliably differentiate anxiety and depression constructs in individuals with TBI. Our hypotheses were guided by common heuristics in the literature for interpreting bifactor statistical indices^34^ and previous findings from non-TBI samples^31–34^. We formed three hypotheses:

### Hypothesis 1

The HADS latent variable structure in TBI would comprise a dominant general distress factor (≥ 80% of systematic variance in HADS total scores explained by general factor) and the specific anxiety and depression factors would have low reliability (< 50% residual variance in subscale scores accounted for by corresponding specific factor).

### Hypothesis 2

There would be minimal bias in treating the HADS as a unidimensional measure compared with using the two traditional subscales (≥ 70% of common variance across item set and ≥ 70% of item intercorrelations attributable to general factor).

### Hypothesis 3

Observations of a dominant general distress factor and unidimensionality of the HADS may be a consequence of high psychiatric comorbidity or presence of more generalized emotional distress in our sample of participants with TBI, not the psychometric properties of the HADS per se^5,15^. To address this issue, we examined relationships between HADS scores and DSM-IV anxiety and depressive disorders diagnosed via semi-structured clinical interview. We hypothesized that the HADS subscales would be unable to discriminate between formal diagnoses of anxiety and depressive disorders.

## Methods and results

The methods and results are presented in a combined section to improve the flow and assist the reader in understanding the statistical concepts.

### Methods overview

Participants were sampled from a larger longitudinal head injury outcomes study^36^. They were recruited from consecutive inpatient TBI admissions to Epworth HealthCare, a hospital in Victoria, Australia that provides comprehensive inpatient and outpatient rehabilitation to 30–50% of all head injuries in the state. Treatment is provided through a no-fault accident compensation scheme that is accessible regardless of socioeconomic status. As a result, TBIs in this cohort are predominantly sustained in motor vehicle collisions. Patients with TBI treated at Epworth HealthCare were approached on the inpatient ward to participate in follow-up research interviews, and written informed consent was obtained from participants or guardians. Approval was obtained from Monash University and Epworth HealthCare ethics committees. All methods were performed in accordance with the relevant guidelines and regulations.

This investigation analyzed data from patients enrolled in the longitudinal study between 1998 and 2019. A subsample of patients admitted between 2005 and 2016 also consented to take part in a smaller post-TBI prospective psychiatric disorders study by clinical interview^2,37^. The current investigation focused on data obtained one year after the injury in both studies. We selected this time-point because: (1) this time-point provided the most data; (2) previous research indicates that this phase of TBI recovery is associated with a particularly high vulnerability for anxiety and depression^37,38^. All participants completed the HADS at this time-point, which was used to address Hypotheses 1 and 2. The subsample who took part in the prospective psychiatric study also completed the Structured Clinical Interview for DSM-IV Axis I Disorders, Research Version (referred to simply as ‘the SCID’ henceforth) contemporaneously with the HADS, which was used to address Hypothesis 3. The SCID and HADS were administered over the phone for the subsample of participants in the psychiatric study. Research interviews for the rest of the sample were also usually conducted over the phone, but the HADS was mailed out if participants preferred.

### Participants

Participants in this investigation had sustained a moderate-severe TBI as defined by the Mayo Classification System^39^, characterized by at least one of the following: worst score of ≤ 12 on the Glasgow Coma Scale^40^ in the first 24 h after injury; ≥ 1 day of post-traumatic amnesia (PTA), measured prospectively using the Westmead PTA Scale^41^; and intracranial abnormality detected on computed tomography (CT) scan. Other inclusion criteria were 16 years or older at the time of injury and sufficient cognitive and English ability to participate, as determined by a clinician. Participants with mild TBI, penetrating head injury, or pre- or post-injury diagnosis of another serious neurological condition (e.g., stroke, brain tumor, neurodegenerative disease) were excluded. However, participants were not excluded on the basis of multiple TBIs or any psychiatric, behavioral, or learning difficulties.

We extracted a data set of 951 individuals, admitted to Epworth HealthCare between 1998 and 2019. After exclusions due to incomplete HADS data or ineligibility, the final sample size consisted of 874 participants, including 189 who also completed the SCID in the prospective psychiatric study. As shown in Table 1, participants were mostly middle-aged males with a high-school education, and the majority of TBIs were sustained in car or motorcycle collisions. Detailed data on ethnicity are not available but the overall cohort from which the sample was drawn is over 90% White, and 9.64% of participants had a non-English-speaking background. The subsample who completed the SCID did not differ significantly from the rest of the sample in demographic or injury-related characteristics except for age (*p* = 0.03), with the subsample being approximately 2 years younger. However, this age difference was negligible in size (Cohen’s *d* = 0.18), and there were no significant associations between age and HADS scores (total score: *p* = 0.87; anxiety subscale: *p* = 0.11; depression subscale: *p* = 0.19).

**Table 1:** Demographic, pre-injury, and injury characteristics of sample.

	*n* (%)	M (SD)	Range
Sex			
Male	643 (73.57%)		
Female	231 (26.43%)		
Age (years)		40.54 (18.14)	17.21–91.01
Education level at injury (years)		11.78 (2.54)	3–22
Employment			
Employed/studying	336 (46.93%)		
Not in labor force	81 (11.31%)		
Unable to work/unemployed	299 (41.76%)		
Relationship status			
Single/never married	312 (43.15%)		
Married or in de facto relationship	320 (44.26%)		
Divorced or separated	74 (10.24%)		
Widowed	17 (2.35%)		
Accommodation			
Independent	661 (90.92%)		
Family/others but need supervision	58 (7.98%)		
Hospital/supported accommodation	7 (0.96%)		
Other	1 (0.14%)		
Non-English speaking background			
No	723 (89.70%)		
Yes	83 (10.30%)		
Injury cause			
Car collision	460 (52.63%)		
Motorcycle collision	126 (14.42%)		
Bicycle collision	47 (5.38%)		
Hit by vehicle as pedestrian	133 (15.22%)		
Fall	51 (5.84%)		
Other	57 (6.52%)		
TBI severity—PTA (days)		14 (5–28)^a^	0–163
Mild: PTA ≤ 1 days	97 (11.33%)		
Moderate: PTA > 1 day and < 7 days	161 (18.81%)		
Severe: PTA ≥ 7 days	598 (69.86%)		
Worst 24-h GCS		9.03 (4.35)	3–15
Acute intracranial findings on CT			
Abnormality detected	787 (90.36%)		
Normal	84 (9.64%)		

Age, employment status, relationship status, and accommodation are at one year post-injury. Data were available for 874/874 (sex; age at injury; injury cause), 843/874 (education level), 716/874 (employment), 723/874 (relationship status), 727/874 (accommodation), 806/874 (non-English speaking background), 856/874 (PTA), 820/874 (GCS), and 871/874 (CT).

*TBI* traumatic brain injury, *PTA* post-traumatic amnesia, *GCS* Glasgow Coma Scale, *CT* computed tomography.

^a^Median (interquartile range).

### Measures

All 874 participants completed the HADS^15^, a self-report measure of anxiety and depression symptoms experienced during the last week*.* Designed for hospital settings, the HADS was constructed to minimize the inclusion of symptoms of anxiety and depression that overlap with direct physiological consequences of medical conditions such as TBI (e.g., sleep disturbance, concentration difficulties). The HADS consists of seven items that form an anxiety subscale and another seven items that address symptoms of depression. Each item is rated from 0 to 3 with variable response labels (subscale score range = 0–21; total score range = 0–42). Some items are reverse-scored, and responses are summed, with higher scores indicative of greater anxiety and depression symptoms*. S*ubscale scores ≥ 8 are considered clinically significant. Descriptive statistics for the HADS are presented in Table 2. Large proportions of participants reported clinically significant anxiety (40.50%) and depression symptoms (33.64%). More participants scored within the clinically significant range on both HADS subscales (26.43%) than either the anxiety (14.07%) or depression subscale alone (7.21%). The HADS subscale scores were strongly positively correlated, *r*(872) = 0.71, *p* < 0.001.

**Table 2:** Descriptive statistics for HADS (*n* = 874).

HADS score	M	SD	Range
Anxiety	6.79	4.90	0–21
Depression	5.85	4.80	0–20
Total	12.64	8.98	0–40

Possible range of HADS subscale scores is 0–21. Total score can range from 0 to 42.

*HADS* Hospital Anxiety and Depression Scale.

Some participants (*n* = 189) also completed the SCID^42^, a semi-structured clinical interview assessing DSM-IV criteria for Axis I psychopathology, including depressive, anxiety, and adjustment disorders. The SCID has been used extensively after moderate-severe TBI, with high inter-rater reliability (κ ≥ 0.80) achieved^37,38^. Our SCID procedures are detailed elsewhere^2^. Briefly, the SCID was administered at one year post-TBI to assess the presence of DSM-IV Axis I disorders between 6 and 12 months after injury. The SCID was administered by clinician-researchers with specialized training in the interview schedule and doctoral training in clinical neuropsychology. Where possible, the SCID was also corroborated by an informant nominated by the participant with TBI. A consensus diagnosis approach was used where the researchers shared their diagnostic impressions based on all available information, including the individual’s self-report, the informant’s report (if available), and information from medical records.

In addition to measures of emotional distress, the degree of disability experienced after TBI was characterized using the Glasgow Outcome Scale—Extended (GOSE), a semi-structured interview with high inter-rater reliability (κ = 0.85)^43^. The examiner rates the disruption to occupational, social, and leisure activities post-injury, with scores of 7–8 indicating ‘good recovery’ (e.g., resumption of normal life but may have minor neurological deficits), 5–6 indicating ‘moderate disability’ (e.g., have some disability but are able to look after themselves), and 3–4 indicating ‘severe disability’ (e.g., dependent on daily support. Of the 874 participants, 752 completed the GOSE. Approximately one-third (34.57%) had made a good recovery one year after their TBI, while two-thirds had ongoing moderate (50.66%) or severe disability (14.76%).

### Data analysis

R software, version 4.1.0^44^, was used. To evaluate the latent variable structure of the HADS, two confirmatory factor models were estimated using the R package *laavan*^45^: a symmetrical bifactor model and a unidimensional model. In the bifactor model, all HADS items loaded on a general factor and one of two specific factors representing the traditional anxiety and depression subscales. Factors were set to be orthogonal. In the unidimensional model, all HADS items loaded only on a general factor. We present the results obtained using the robust maximum likelihood estimator (MLR), providing corrections to standard errors and test statistics. Although the weighted least squares means and variance adjusted (WLSMV) estimator was considered due to the ordinal response scale of the HADS, it resulted in a negative estimated variance (an implausible value) in one of the factor models, casting doubts about the WLSMV estimation method. The MLR and WLSMV estimators produced similar results outside of this one model.

In contrast to previous factor analytic studies, we did not consider traditional model fit indices for the bifactor model in this study. These indices are biased in favor of bifactor models due to their relatively large number of parameters and consequent flexibility to overfit noise^46–50^. Instead, we employed *bifactor statistical indices* (e.g., omega hierarchical coefficients), using the R package *BifactorIndicesCalculator*^51^, as a robust alternative to evaluate the latent variable structure of the HADS^30,34^. However, traditional model fit indices were considered for the unidimensional model to further assess the degree of unidimensionality of the HADS. We used the root mean squared error of approximation (RMSEA) to quantify absolute unidimensional fit of the unidimensional model (values < 0.06 indicate close fit), and for incremental fit, the Comparative Fit and Tucker–Lewis Indices (CFI and TFI, respectively; values > 0.95 indicate close fit)^52^. Simulation studies indicate that these fit cut-offs rarely misclassify true unidimensional models as incorrect^53^. To appraise the coherence and meaningfulness of factors in both the bifactor and unidimensional models, we also considered the direction of factor loadings (all expected to be positive) and their salience (absolute λ ≥ 0.32 considered adequate)^54^: absolute λ ≥ 0.71 = ‘excellent’, λ ≥ 0.63 = ‘very good’, λ ≥ 0.55 = ‘good’, λ ≥ 0.45 = ‘fair’, λ ≥ 0.32 = ‘poor’, λ ≤ 0.32 = ‘very low’ and inadequate.

### Hypothesis 1: testing the dominance of a general distress factor

**Figure 2:**
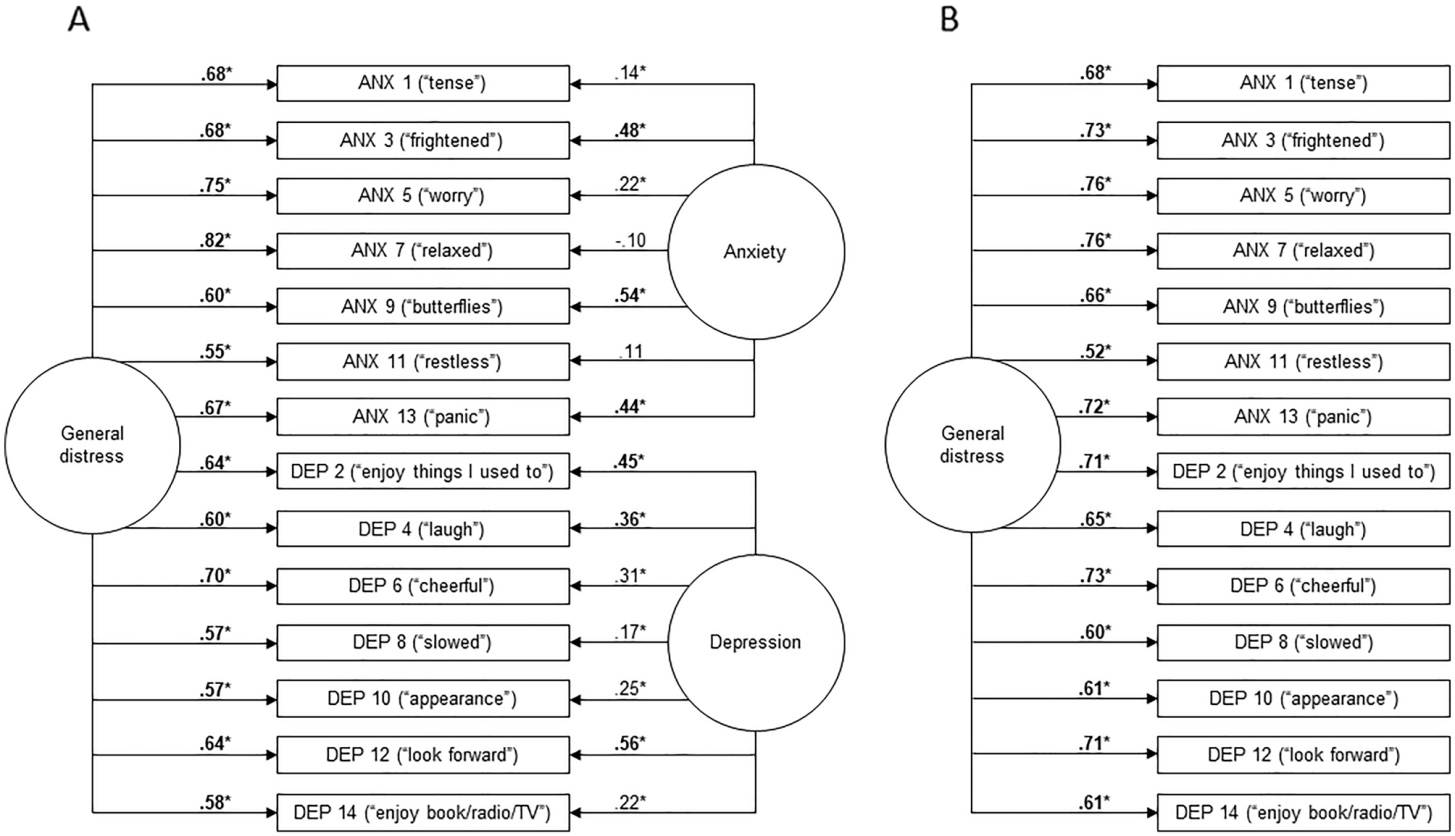
Path diagram of the confirmatory symmetrical bifactor (**A**) and unidimensional models (**B**) of the HADS (*n* = 874). Factor loadings considered at least adequate (absolute λ ≥ 0.32) are shown in boldface. Significant factor loadings are indicated with an asterisk. All factor loadings were significant (all *p*s ≤ 0.001) except for the loadings of Items 7 and 11 on the specific anxiety factor (*p*s > 0.05). *ANX* anxiety subscale item, *DEP* depression subscale item.

Figure 2A presents the confirmatory symmetrical bifactor model of the HADS. All items had stronger loadings on the general factor (rated as ‘good’ to ‘excellent’) than on their specific factor (rated ‘very poor’ to ‘good’), meaning that they were better indicators of general distress than specific anxiety or depression. Of the 14 specific factor item loadings, only 6 were considered adequate (absolute λ ≥ 0.32). Moreover, two anxiety subscale items (Items 7 “relaxed” and 11 “restless”) did not significantly load on the specific anxiety factor (*p* ≥ 0.05). These results suggest that, when a general distress factor is included, the coherence and meaningfulness of the specific HADS anxiety and depression factors are questionable.

We computed bifactor statistical indices, specifically, omega hierarchical reliability coefficients, to formally quantify the dominance of the HADS general distress factor^30^. *Omega hierarchical* (omegaH) refers to the proportion of systematic variance in HADS total scores captured by the general factor. *Omega hierarchical subscale* (omegaHS) measures the proportion of systematic variance in HADS anxiety or depression subscale scores uniquely attributable to the corresponding specific factor, *after partialling out variance due to the general factor*. OmegaH and omegaHS values < 0.50 are considered indicative of insufficient reliability for interpretation of the factor (general or specific, respectively)^55,56^. A dominant general factor is indicated by omegaH ≥ 0.80 and comparatively small omegaHS values (< 0.50). We obtained an omegaH value of 0.84, indicating that 84% of the systematic variance in HADS total scores was captured by the general factor. After partialling out variance due to the general factor, the anxiety and depression factors accounted for only 12% and 20% of residual variance in the anxiety and depression subscale scores, respectively (omegaHS_Anxiety_ = 0.12; omegaHS_Depression_ = 0.20). These results suggested the presence of a dominant general distress factor underlying the HADS data structure, with low reliability of the specific factors above and beyond the general factor.

### Hypothesis 2: testing the unidimensionality of the HADS

Given a dominant general distress factor, we next sought to establish whether the HADS was “unidimensional enough” in individuals with TBI to justify using the total score instead of the subscale scores^30^. This was achieved by computing additional bifactor statistical indices. *Explained common variance* (ECV) calculates the proportion of variance common to the HADS item set that is specific to the general factor. *Percentage uncontaminated correlations* (PUC) measures the proportion of correlations between items from different HADS subscales and therefore that are “uncontaminated by multidimensionality”, reflecting only variance from the general factor. ECV and PUC of ≥ 0.70 would indicate that the HADS is “essentially unidimensional”, with the specific factors only representing noise in the data rather than meaningful subscales^30^. We also calculated the ECV for each specific factor (S-ECV) to quantify its uniqueness (ideally S-ECV ≥ 0.70 for a unique specific factor) and the *value-added ratio* (VAR) for each HADS subscale, determining whether the observed subscale scores (including measurement error) accounted for more variance in the true subscale scores (without measurement error) than the observed total score (VAR > 1.1 required for added value of subscales)^57,58^. The VAR values were computed using the Haberman method^59^ via the R package *subscore*^60^. In the case of non-meaningful specific factors, we would expect minimal bias in fitting a unidimensional model to the HADS—that is, the model parameter estimates should change little when removing the specific factors of anxiety and depression. This can be quantified through the *average relative parameter bias* (ARPB), the mean difference in item loadings on the general factor between the bifactor and unidimensional models (< 10% difference is ideal for unidimensionality)^61^.

Our analysis revealed that 78% of the variance common to the HADS item set was attributable to the general distress factor (ECV = 0.78; ≥ 0.70 ideal for unidimensionality). The little remaining common variance was distributed across the specific anxiety (S-ECV_Anxiety_ = 0.11) and depression factors (S-ECV_Depression_ = 0.12). The subscale scores of anxiety and depression did not add value above and beyond the total score (VAR_Anxiety_ = 1.05 and VAR_Depression_ = 1.03). Fitting a unidimensional model to the HADS (see Fig. 2B) resulted in only a 7% change in the loadings on the general factor compared to the bifactor model containing the specific anxiety and depression factors (ARBP = 0.07; < 0.10 ideal for unidimensionality). The loadings in the unidimensional model were all positive and rated ‘good’ to ‘excellent’ in strength, indicating a coherent and meaningful general factor. However, only 54% of correlations between HADS items reflected variance on the general factor (PUC = 0.54; ≥ 0.70 ideal for unidimensionality), with 46% of item intercorrelations “contaminated by multidimensionality”. In addition, absolute fit of the unidimensional model was poor (robust RMSEA = 0.11; < 0.06 ideal), as was incremental fit (robust CFI = 0.87; robust TLI = 0.84; > 0.95 ideal).

These results suggested that the HADS item responses were not closely aligned with a unidimensional model (as indicated by PUC < 0.70 and poor unidimensional model fit), but further, sources of multidimensionality in the data were not adequately captured by the traditional anxiety and depression subscales (as indicated by low S-ECV and VAR values). Therefore, we analyzed the bifactor model at the item level to gain more insight (see Supplemental Table S1 for full statistics). We calculated the ECV and relative parameter bias for each individual item (I-ECV and RPB, respectively) to assess their contribution to multidimensionality in the data structure. Although there are no established cut-offs, we identified any items that had both I-ECV < 0.85 and RBP ≥ 0.10 as having a meaningful association with their specific factor^61–63^. This would mean that more than 15% of variance at the item level was attributable to the specific factor and the item’s loading on the general factor would change by at least 10% when the specific factor was removed from the model. Only two HADS items were found to be meaningfully associated with their specific factor, and they belonged to the traditional depression subscale: Items 2 “enjoy things I used to” and 12 “look forward”. Although these items had the strongest loadings on the specific depression factor (λ = 0.42 and 0.56, respectively), they were still stronger indicators of general distress (both λ = 0.64). The content of these items suggest that they might reflect symptoms of anhedonia, but they could also be influenced by the direct consequences of TBI or the experience of disability.

### Sensitivity analyses

We conducted three sensitivity analyses to examine the influence of the degree of disability experienced by participants, non-English speaking background, and time post-injury on the bifactor analysis results. First, we repeated the factor analyses separately with participants who had good recovery at one year post-injury (GOSE score = 7 or 8) and those with ongoing moderate or severe disability (GOSE score = 3–6). This was done to investigate whether the latent variable structure of the HADS was affected by TBI symptoms or the experience of disability. Second, we re-estimated the factor models using only native English speakers, as the HADS is a verbally mediated measure. Third, there is evidence suggesting that anxiety and depression after TBI follow different trajectories, with the latter being more persistent^37^. Therefore, we explored the latent variable structure of the HADS in a more chronic period post-TBI by repeating the analysis at five years post-injury. This analysis relied on participants from the one-year dataset who had also responded to all items of the HADS at the five-year post-injury research interview by the time of data extraction. The results of these sensitivity analyses, presented in Table 3, suggested that the degree of disability experienced by participants, non-English speaking background, and time post-injury did not significantly influence the latent variable structure of the HADS, as evidenced by the highly similar model- and factor-level bifactor statistical indices.

**Table 3:** Results of sensitivity analyses.

	Original analysis (*n* = 874)	Participants with good recovery (*n* = 260)	Participants with moderate or severe disability (*n* = 492)	Only native English speakers (*n* = 723)	Participants at 5 years post-injury (*n* = 395)
OmegaH/HS					
General distress	0.84	0.80	0.83	0.83	0.83
Anxiety	0.12	0.11	0.14	0.15	0.17
Depression	0.20	0.26	0.20	0.20	0.18
ECV					
General distress	0.78	0.74	0.75	0.76	0.75
Anxiety	0.11	0.11	0.13	0.12	0.14
Depression	0.12	0.16	0.12	0.12	0.11
PUC	0.54	0.54	0.54	0.54	0.54
ARPB	0.07	0.08	0.07	0.07	0.08

*OmegaH* omega hierarchical coefficient (*HS* coefficient for subscales), *ECV* explained common variance, *PUC* percentage uncontaminated correlations, *ARPB* average relative parameter bias.

### Hypothesis 3: examining relationships between HADS scores and formal psychiatric diagnoses

To clarify if the bifactor analysis results were due to the psychometric properties of the HADS or instead high rates of psychiatric comorbidity in our sample of individuals with TBI, we conducted secondary analyses in a subsample of 184 participants who also completed the SCID. Since individuals with TBI often have comorbid anxiety and depressive disorders^1,3^, scores on the HADS anxiety and depression subscales may be highly correlated in this population, even if they are measuring discrete constructs^5,15^. This could exaggerate the bifactor statistical indices in favor of the general factor. We examined relationships between HADS scores and SCID-diagnosed DSM-IV anxiety and depressive disorders, and compared individuals diagnosed by a trained clinician-researcher via structured interview to be experiencing *only* anxiety or depression. This allowed us to minimize the influence of psychiatric comorbidity in our psychometric evaluation of the HADS.

We conducted three analyses of covariance (ANOVAs; see Supplemental Tables S2–S5 for full statistics) to compare HADS scores between four groups of participants: (1) 108 participants (58.70%) who were not diagnosed with any DSM-IV Axis I disorder (‘*NO DX*’ group); (2) 19 participants (10.33%) diagnosed with one or more anxiety disorders *but no depressive disorder* (‘*ANX ONLY*’ group); (3) 23 participants (23.50%) diagnosed with a depressive disorder *but no anxiety disorder* (‘*DEP ONLY*’ group); and (4) 34 participants (18.48%) diagnosed with *both* one or more anxiety disorders and a depressive disorder (‘*COMORBID*’ group). Adjustment disorders characterized by depressed mood or anxiety were treated as depressive and anxiety disorders, respectively. Participants diagnosed with an adjustment disorder characterized by *mixed* anxiety and depressed mood were categorized under the COMORBID group. Although 189 participants completed the SCID, five participants who met criteria for an Axis I disorder *but not an anxiety, depressive, or adjustment disorder* (with anxiety and/or depressed mood) were excluded from this secondary analysis (*n* = 184 analyzed). A significantly greater proportion of participants in the DEP ONLY group (86.67%) were single compared with the NO DX (51.95%) and ANX ONLY groups (43.75%). However, relationship status was not significantly associated with HADS scores (total score: *p* = 0.90; anxiety subscale: *p* = 0.87; depression subscale: *p* = 0.71).

Significant ANOVAs were followed by post-hoc comparisons using Tukey’s honest significant test (HSD) to determine which SCID diagnostic groups differed in their HADS scores. Non-significant differences (*p* ≥ 0.05, adjusted for multiple comparisons) in HADS subscales scores between the ANX ONLY and DEP ONLY groups would suggest that the HADS cannot reliably differentiate between anxiety and depression. In addition to testing for statistically significant *differences*, we also assessed whether the observed differences between these diagnostic groups were small enough to be considered statistically *equivalent* or trivial. We used concepts of the ‘minimal clinically important difference’ (MCID), which is the smallest difference on a measure believed to be clinically relevant^64^, and equivalence testing^65^. Although MCIDs for the HADS have not been studied in the context of TBI specifically, research on the HADS in patients with cardiovascular or lung disease suggests MCID estimates between 1.7 and 2.5 raw points for each subscale^66–69^. We performed equivalence testing using the two one-sided tests (TOST) procedure^65^ to establish whether we could declare the absence of a difference of at least two raw points on the HADS subscales between the ANX ONLY and DEP ONLY groups. Statistical equivalence of the HADS subscales between these diagnostic groups (and therefore a lack of discriminant validity) would be supported if *p* < 0.05 for both the lower and upper equivalence bounds of − 2 and + 2 points, respectively. Recognizing the inherent limitation of relying solely on *p*-values with small group sizes, we also employed standardized effect sizes to interpret differences in HADS scores between groups: Cohen’s *d* < 0.20 indicated a ‘negligible’ difference, Cohen’s *d* = 0.20–0.49 indicated a ‘small’, clinically meaningful difference; Cohen’s *d* = 0.50–0.79 indicated a ‘medium’ difference; and Cohen’s *d* ≥ 0.80 indicated a ‘large’ difference^70^.

**Figure 3:**
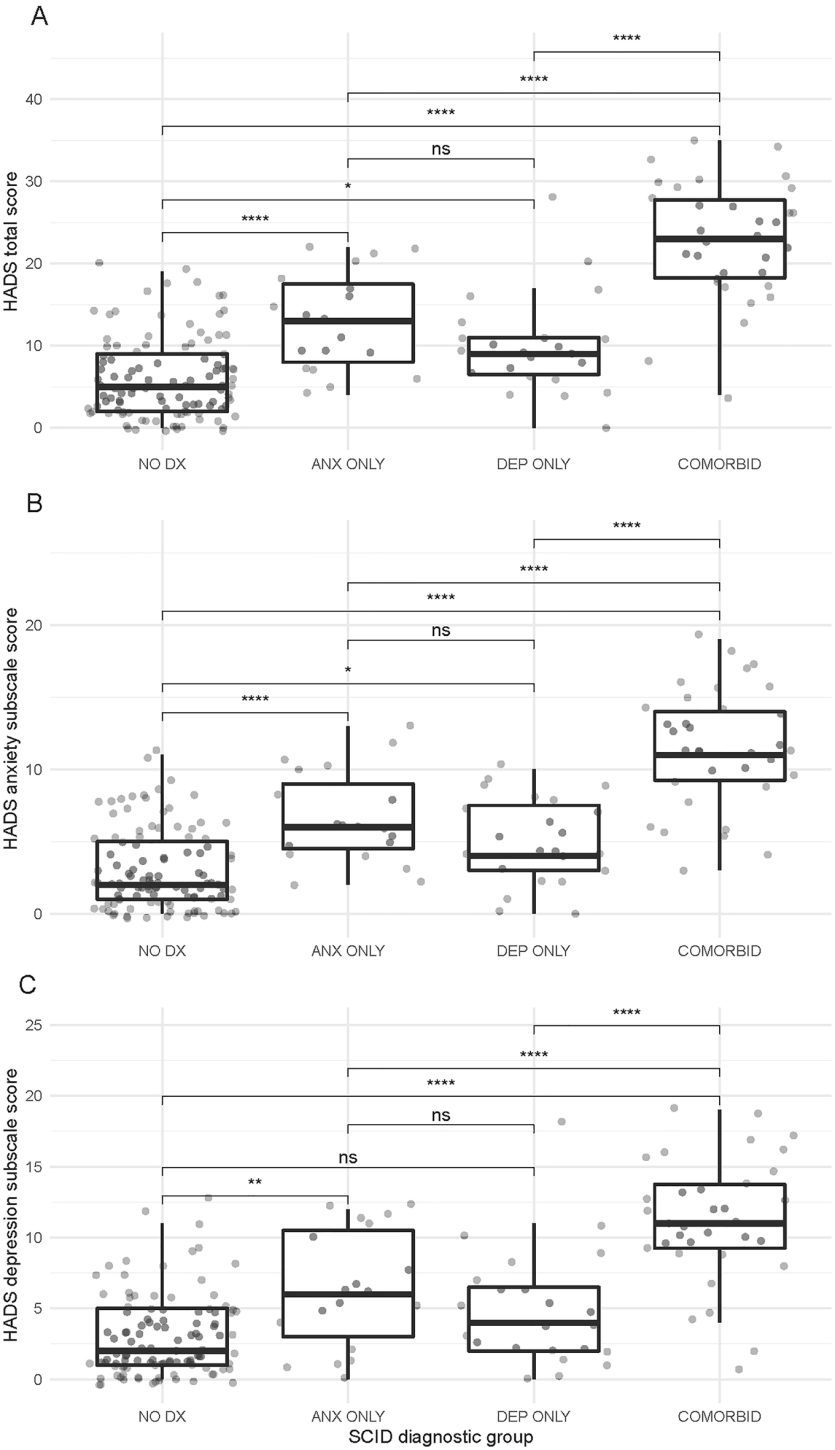
Box plots showing the distributions of HADS total (**A**), anxiety subscale (**B**), and depression subscale scores (**C**) between four psychiatric diagnostic groups (total *n* = 184). Dots represent the HADS scores of individual participants. Group means were compared using Tukey’s honest significant difference, with *p*-values adjusted for multiple comparisons. *HADS* Hospital Anxiety and Depression Scale, *SCID* Structured Clinical Interview for DSM-IV Axis I Disorders. *NO DX* no SCID diagnosis of any Axis I disorder (*n* = 108). *ANX ONLY* SCID diagnosis of one or more anxiety disorders *but no depressive disorder* (*n* = 19). *DEP ONLY* SCID diagnosis of a depressive disorder *but no anxiety disorder* (*n* = 23). COMORBID = SCID diagnoses of *both* one or more anxiety disorders and a depressive disorder (*n* = 34). *Adjusted *p* < 0.05, **adjusted *p* < 0.01, ***adjusted *p* < 0.001, ****adjusted *p* < 0.0001, *ns* non-significant (adjusted *p* ≥ 0.05).

As shown in Fig. 3, individuals with COMORBID diagnoses had significantly higher HADS total, anxiety, and depression scores than those with DEP ONLY or ANX ONLY (adjusted *p*s < 0.001; large differences of Cohen’s *d* = 1.18–1.96). On the other hand, we found no statistically significant differences in HADS scores between participants with ANX ONLY and those with DEP ONLY (adjusted *p*s = 0.33–0.62). However, at the same time, we could not declare that these groups had statistically *equivalent* scores. Specifically, we could not reject a true difference where the ANX ONLY group had scored meaningfully higher than those with DEP ONLY not only on the HADS anxiety subscale (*p* = 0.35) *but also the depression subscale (p* = 0.30). In line with this, the ANX ONLY group had clinically significant higher HADS scores as indicated by small-to-medium standardized effect sizes (Cohen’s *d* = 0.31–0.52). The DSM-IV contains a greater number and breadth of individual anxiety disorders that can co-occur (mean number of individual diagnoses in ANX ONLY group = 1.63 vs. 1.29 for DEP ONLY). Potentially, this led to greater reported distress symptomatology on the HADS in the ANX ONLY group. Nonetheless, this analysis did not support the discriminant validity of the HADS subscales since the ANX ONLY group demonstrated higher depression subscale scores.

## Discussion

This study used novel bifactor statistical indices to evaluate whether the HADS can reliably differentiate anxiety and depression in individuals with TBI. Hypothesis 1 of a dominant general distress factor was supported, with this factor accounting for 84% of the systematic variance in HADS total scores. When holding the general factor constant, the HADS did not reliably differentiate specific anxiety and depression constructs. Hypothesis 2 that the HADS would be essentially unidimensional was partially supported. The general factor was much stronger in explaining variance common to the HADS item set than the specific factors and the subscale scores did not add value over the total score. Yet, nearly half of the item intercorrelations did not reflect the general factor’s influence, and a unidimensional model demonstrated poor fit. Two individual items from the depressions scale were identified as sources of multidimensionality, but they were still stronger indicators of general distress than the specific depression factor. Further, there was minimal bias in treating the HADS as a unidimensional measure, with loadings on the general factor differing by only 7% on average between the bifactor and unidimensional models. The bifactor analysis results were consistent in sensitivity analyses accounting for the degree of disability experienced by participants, non-English-speaking background, and time post-injury. Lastly, Hypothesis 3 was also supported, as the HADS subscales did not clearly discriminate between formal SCID diagnoses of DSM-IV anxiety and depressive disorders.

At first glance, our findings may appear somewhat inconsistent with previous research regarding the dimensionality of the HADS for individuals with TBI. Previous studies have suggested that two- and three-factor models fit the HADS best in this population^20–22^. However, these studies did not employ bifactor modeling, which *completely* partitions systematic variance into components due to a general factor versus specific factors. Schӧnberger and Ponsford^21^ modeled a higher-order negative affect factor, but this factor was set to only have a direct influence on four items; its contributions to the remaining items were mediated via lower-order factors of anxiety and depression. As such, this model did not tease apart the unique contributions of the higher- and lower-order factors^35^. Our study found that the specific factors of anxiety and depression of the HADS were no longer reliable or conceptually meaningful after completely partialing out variance attributable to a higher-order factor of general distress. Despite differences in dimensionality assessment, the previous factor analytic studies alluded to a dominant general distress factor, as they observed strong positive correlations between factors of negative affect, anxiety, and depression^20–22^. Additionally, our study is not the first to demonstrate that the HADS subscale scores poorly map onto their corresponding psychiatric diagnoses in individuals with TBI^5,18,19^.

The components of psychopathology driving item response patterns on the HADS can be interpreted in relation to structural models of anxiety and depression. Empirical evidence is increasingly suggesting that the structure of psychopathology comprises numerous *dimensions* (not categories) organized *hierarchically*. Higher-order dimensions are thought to account for comorbidity among lower-order dimensions such as anxiety and depression^8,26,71–73^. One of the earliest models of this type is Clark and Watson’s^26^ Tripartite Model of Anxiety and Depression, which proposes a general component—*negative affect*—common to anxiety and depression, alongside more specific factors of *hyperarousal* (unique to anxiety) and *low positive affect* or *anhedonia* (unique to depression). More recently, the Hierarchical Taxonomy of Psychopathology (HiTOP)^8,74^ was developed as a dimensional-hierarchical model based on consensus of empirical evidence that attempts to map the full range of psychopathological problems. Regarding anxiety and depression, HiTOP includes a *distress* subfactor (covering symptoms of major depressive, generalized anxiety, and trauma- and stressor-related disorders) and a *fear* subfactor (covering symptoms of panic, phobias, and obsessive–compulsive disorders), both of which are subsumed by a higher-order *internalizing* spectrum. From this perspective, we found that scores on the HADS subscales do not reliably parse components unique to anxiety and depression but instead predominately reflect variance on a higher-order factor representing a component common to *both* these psychopathological syndromes—‘negative affect’, ‘general distress’, ‘internalizing psychopathology’, etcetera. The two depression HADS items highlighted in this study as possessing a meaningful association with the depression specific factor above and beyond the general factor may be tapping into more unique structural components of depression—low positive affect/anhedonia—as articulated in the Tripartite Model. However, these items may have also introduced multidimensionality due to being influenced by the direct consequences of TBI or the experience of disability.

TBI can be a significantly life-altering injury, resulting in the loss of previously valued roles, relationships, and activities, as well as changes to one’s sense of self^75–77^. Especially in the early years post-injury, individuals with TBI may experience generalized emotional distress, such as grief, anger, fear, and hopelessness, as they try to accept, cope with, and adjust to a life-altering injury^5^. Concordantly, this population presents with a wide range of emotional symptoms that can lead to formal diagnoses of both anxiety and depressive disorders, with reported comorbidity rates as high as 77%^3^. However, by leveraging psychiatric diagnostic data obtained through semi-structured clinical interviews, we tried to address this issue, demonstrating that issues with the HADS anxiety and depression subscales remained when comparing individuals with formal diagnoses of *only* anxiety and those with *only* depression. Additionally, the omega hierarchical coefficients obtained in our sample of individuals with TBI (omegaH = 0.84, omegaHS_Anxiety_ = 0.12, omegaHS_Depression_ = 0.20) were similar to those reported in other populations (omegaH = 0.72–0.84, omegaHS_Anxiety_ = 0.04–0.13, omegaHS_Depression_ = 0.13–0.48)^31–34^. All the previous studies we are aware of that used bifactor statistical indices have also concluded that a dominant general distress factor and specific anxiety and depression factors of low reliability are present within the HADS latent variable structure. Similar results have been obtained in general community samples, where rates of psychiatric comorbidity are expected to be lower when compared with individuals with chronic illnesses^32,34,78^. Therefore, we believe our results are not confounded by the issue of psychiatric comorbidity or unique to TBI or medical populations, but instead reflect the psychometric properties of the HADS.

Our research has significant implications. Our study is the first to use bifactor statistical indices in individuals with TBI to examine the latent variable structure of the HADS more closely. Conceptually, our findings provide some preliminary evidence for the generalizability of structural models of anxiety and depression symptoms to TBI as described above, adding to previous research seeking to understand the complex relationship between anxiety and depression in TBI and other neurological populations^23–25,79,80^. Practically, our findings argue against the status quo of scoring and interpreting the traditional HADS subscales. Our results suggest that the anxiety and depression subscales largely tap into the same, single underlying latent variable. On the other hand, the total score reflects variance on a coherent and meaningful general factor that is sensitive to overall psychiatric burden. As such, whilst they will understandably examine endorsement of individual symptoms to inform treatment, clinicians and researchers should exercise caution in interpreting the individual HADS subscales and instead consider using the total score as a more valid measure of general distress in individuals with TBI. At present, there is limited guidance on interpreting the HADS total score. However, normative data is available for the total score^81^, allowing for standardization of this score (e.g., *T*-scores, percentiles) and interpretation using graded clinical cut-offs of mild, moderate, and severe (e.g., *T* = 55, 65, and 75) as recommended in the psychiatric literature^82^. Future research will need to thoroughly investigate the clinical utility of using the HADS total score in individuals with TBI (e.g., identifying cut-offs with high sensitivity and specificity for detecting emotional disorders).

Our results suggest that there may be some multidimensionality in the HADS data structure that the traditional subscales are unable to capture. It is important to pay close attention to the specific symptoms being assessed. Reliably extracting different symptom dimensions from the HADS may require adding or removing items^31^, and although the HADS minimizes the inclusion of symptoms that overlap with medical conditions, concerns remain about whether its latent variable structure is impacted by TBI symptoms or the experience of disability. By comparing the item content of the HADS and the more comprehensive SCID, we can identify symptoms that could be added to potentially improve the HADS’ ability to differentiate between different forms of psychopathology, such as depressed mood, changes in appetite or weight, self-deprecation, indecisiveness, suicidality, traumatic intrusions, traumatic avoidance, and context-specific fear (e.g., social anxiety). However, TBI-related influences on these symptoms would need to be considered. Future studies could supplement the HADS items with items from other measures^83^ or use network analysis to investigate the relationships between individual HADS symptoms^12^. Further, the HADS assesses symptoms experienced in the past week, which may lead to an averaging or ‘smoothing out’ of distinct affective states (e.g., sadness, fear) sequentially experienced by individuals over time. While measures of general distress averaged over a period of time provide valuable information and are widely used in clinical practice, exploring the temporal dynamics of anxiety and depressive symptoms experienced in the moment by an individual, for example, through ecological momentary assessment (EMA), could be a fruitful direction for future research^84,85^.

We acknowledge that the HADS was not designed as a diagnostic tool to discriminate anxiety and depressive disorders^19,86^, and clinicians are likely instead focused on documenting and treating the specific symptoms experienced by the individual^11,87^. Therefore, our findings may have modest implications for clinicians at this time, simply serving to affirm their current practices. The study has more immediate and significant implications from a research perspective, as researchers frequently use the HADS subscales to differentially measure the prevalence of anxiety and depression symptoms post-TBI, examine their trajectories, investigate associated factors, and evaluate treatment outcomes^13,14,16,17^.

### Transdiagnostic approaches to psychopathology

The aforementioned Tripartite Model and HiTOP are *transdiagnostic* re-conceptualizations of psychopathology, identifying constructs that transcend the boundaries of traditional diagnostic categories. A transdiagnostic approach may better represent the complex clinical reality that individuals with TBI often present with comorbid psychiatric diagnoses and problems that do not fit neatly into any one diagnostic category^2,11,88^. Evidence from the general population suggests that transdiagnostic dimensions may provide superior reliability and explanatory and predictive power compared to categorical diagnoses^89^, with the potential to improve risk and prognostic models as well as treatments^10^. Clinicians working with individuals with TBI may recognize the benefits of transdiagnostic concepts and deploy these in their work^90,91^. However, their current incorporation of these concepts may be pragmatic and unsystematic, as there is limited research to guide a transdiagnostic approach within this population. It is hoped that future statistical exploration of potential transdiagnostic dimensions will help us to enhance our theoretical understanding and inform and develop treatments for individuals with TBI.

Before a systematic transdiagnostic model can be realized for individuals with TBI, suitable assessment protocols and models need to be developed and validated. Existing psychopathology assessment measures used with individuals with TBI such as the HADS, Depression Anxiety Stress Scales (DASS), and Patient Health Questionnaire Anxiety and Depression Scale (PHQ-ADS) appear too brief to reliably extract separate components of anxiety and depression^92^. Indeed, similar to our findings regarding the HADS, inspection of bifactor models fit to the DASS^93^ and PHQ-ADS^79^ in samples of individuals with TBI suggest specific factors that are unreliable and conceptually ambiguous when accounting for a general distress factor. Assessment measures that are optimal for transdiagnostic research (see for example, the Inventory of Depression and Anxiety Symptoms)^94^ are more comprehensive, including items with varying degrees of generality and specificity to capture both broad spectra and more specific dimensions^8,95^. Multiple non-redundant items addressing the same symptom or trait domain are used to yield more narrow, homogenous dimensions^94^ that can be reliably parsed from a general psychopathology factor^96^.

### Study limitations

The study has several limitations worth noting. First, most participants completed the HADS and SCID over the phone. This may have resulted in lower symptom reporting if participants responded in a socially desirable manner (e.g., due to increased difficulty establishing rapport in the absence of non-verbal communication cues and the physical absence of the interviewer)^14,16,97^. Future research could examine whether our results generalize across different modes of measure administration.

Second, the numbers of participants with a diagnosis of only anxiety or depression were small, limiting the analyses examining the discriminant validity of the HADS subscales. Nonetheless, these results were consistent with our bifactor analysis results obtained in the larger overall sample and with large studies conducted in other populations. Additionally, a significant proportion of the anxiety disorders diagnosed in this study were post-traumatic stress disorder (PTSD) and subsyndromal variants of PTSD. The HADS anxiety subscale was not designed to measure symptoms specific to PTSD such as traumatic intrusions. Nonetheless, PTSD shares numerous cognitive, behavioral, emotional, and physiological features with other anxiety disorders^98^, features which the HADS anxiety subscale appears unable to reliably differentiate from features of depressive disorders.

Third, our sample was representative of the broader population of individuals with TBI with respect to sex, and participants received treatment through a no-fault accident compensation scheme that was accessible regardless of socioeconomic status. However, the generalizability of our findings to other groups, who sustained their injuries predominately via other causes (e.g., falls) or did not receive comprehensive inpatient and outpatient rehabilitation, may be limited. Further, the generalizability of our findings may be constrained to White individuals with TBI. Culturally and linguistically diverse groups may differ with respect to their beliefs about and emotional reactions to TBI, reporting of injury sequalae^99,100^, and the manifestation of psychopathology^101^. Therefore, it is recommended that future studies seek to replicate our findings in ethnically and racially diverse samples of individuals with TBI^102^.

## Conclusions

In conclusion, bifactor modeling is a useful method for evaluating the viability of using total and subscale scores of psychometric instruments. Our study of a large sample of individuals with TBI shows that the HADS cannot reliability differentiate anxiety and depression as discrete constructs. The scale was instead found to predominately measure general distress, justifying the use of the total score and suggesting caution be exercised in interpreting the individual subscales. To study the mental health of individuals with TBI, we propose a shift towards modern, transdiagnostic conceptualizations of psychopathology (e.g., HiTOP). This will require the validation of new assessment approaches for this population that can reliably parse general and specific components of psychopathology.

## Supplementary Information

Supplementary Tables.

## Data Availability

The datasets analysed during the current study are available from the corresponding author on reasonable request.
