# Author Correction: Bifactor analysis of the Hospital Anxiety and Depression Scale (HADS) in individuals with traumatic brain injury

**DOI:** 10.1038/s41598-023-36651-x

**Published:** 2023-06-12

**Authors:** Jai Carmichael, Gershon Spitz, Kate Rachel Gould, Lisa Johnston, Alexia Samiotis, Jennie Ponsford

**Affiliations:** 1 Monash-Epworth Rehabilitation Research Centre, Epworth HealthCare, Melbourne, Australia; 2 School of Psychological Sciences, Turner Institute for Brain and Mental Health, Monash University, Clayton, Australia; 3 Department of Neuroscience, Central Clinical School, Faculty of Medicine, Nursing and Health Sciences, Monash University, Melbourne, Australia

Correction to: *Scientific Reports* 10.1038/s41598-023-35017-7, published online 17 May 2023

The original version of this Article contained an error.

In the Methods and results section, under the subheading ‘Data analysis’,

“Although the weighted least mean and variance adjusted (WLSMV) estimator was considered due to the ordinal response scale of the HADS, it resulted in a negative estimated variance (an implausible value) in one of the factor models, casting doubts about the WLSMV estimation method.”

now reads:

“Although the weighted least squares means and variance adjusted (WLSMV) estimator was considered due to the ordinal response scale of the HADS, it resulted in a negative estimated variance (an implausible value) in one of the factor models, casting doubts about the WLSMV estimation method.”

The original Article has been corrected.

