# Supplementary material for: Bifactor analysis of the Hospital Anxiety and Depression Scale (HADS) in individuals with traumatic brain injury
Source: Sci Rep. 2023 May 17;13:8017. doi: 10.1038/s41598-023-35017-7 (PMC10192445; doi:10.1038/s41598-023-35017-7)
Supplement: Supplementary file 1 — Supplementary Tables. [file 41598_2023_35017_MOESM1_ESM.docx]

**Supplemental Materials for ‘Bifactor analysis of the Hospital Anxiety and Depression Scale (HADS) in individuals with traumatic brain injury’**

**By Carmichael J., Spitz, G., Gould, K. R., Johnston, L., Samiotis, A. & Ponsford, J.**

| HADS item | I-ECV | RPB |
| --- | --- | --- |
| ANX 1 (“tense”) | .96 | .001 |
| ANX 3 (“frightened”) | .67 | .07 |
| ANX 5 (“worry”) | .92 | .01 |
| ANX 7 (“relaxed”) | .99 | .07 |
| ANX 9 (“butterflies”) | .56 | .09 |
| ANX 11 (“restless”) | .96 | .06 |
| ANX 13 (“panic”) | .69 | .08 |
| **DEP 2 (“enjoy things I used to”)** | **.67** | **.10** |
| DEP 4 (“laugh and see funny side”) | .74 | .08 |
| DEP 6 (“cheerful”) | .84 | .05 |
| DEP 8 (“slowed”) | .92 | .05 |
| DEP 10 (“appearance”) | .84 | .07 |
| **DEP 12 (“look forward”)** | **.56** | **.12** |
| DEP 14 (“enjoy book/radio/TV”) | .88 | .04 |

**Table S1.** Item-level bifactor statistical indices (*n*=874).

*Note*. HADS = Hospital Anxiety and Depression Scale. ANX = item from HADS anxiety subscale. DEP = item from depression subscale. I-ECV = percentage of common variance at the item level captured by the general factor. RBP = relative parameter bias, difference in an item’s loading on the general factor between the bifactor and unidimensional models, expressed as a proportion. Boldface items for which both I-ECV<.85 and RPB≥10 were considered as having a meaningful association with their specific factor and therefore as contributing some multidimensionality to the HADS data structure.

| Diagnosis | *n* (%) |
| --- | --- |
| Any Axis I disorder | 76 (41.30%) |
| Any depressive disorder | 54 (29.35%) |
| Major depressive disorder | 37 (20.11%) |
| Depressive disorder not otherwise specified | 17 (9.24%) |
| Any anxiety disorder (one or more) | 50 (27.17%) |
| Panic disorder with agoraphobia | 5 (2.72%) |
| Panic disorder without agoraphobia | 2 (1.09%) |
| Agoraphobia without panic | 2 (1.09%) |
| Specific phobia | 9 (4.89%) |
| Social phobia | 8 (4.35%) |
| Obsessive-compulsive disorder | 3 (1.63%) |
| Post-traumatic stress disorder | 20 (10.87%) |
| Generalized anxiety disorder | 3 (1.63%) |
| Anxiety disorder not otherwise specified | 24 (13.04%) |
| Any adjustment disorder | 5 (2.72%) |
| With depressed mood | 2 (1.09%) |
| With anxiety | 2 (1.09%) |
| With mixed anxiety and depressed mood | 1 (0.54%) |
| Any bipolar disorder | 2 (1.09%) |
| Bipolar disorder not otherwise specified | 2 (1.09%) |
| Alcohol use disorder | 6 (3.26%) |
| Substance use disorder | 7 (3.80%) |
| Amphetamine use | 2 (1.09%) |
| Cannabis use | 3 (1.63%) |
| Hallucinogen use | 1 (0.54%) |
| Any eating disorder | 2 (1.09%) |
| Eating disorder not otherwise specified | 2 (1.09%) |
| ANOVA groups |  |
| NO DX^a^ | 108 (58.70%) |
| ANX ONLY^b^ | 19 (10.33%) |
| DEP ONLY^c^ | 23 (12.50%) |
| COMORBID^d^ | 34 (18.48%) |

**Table S2.** Frequency of SCID Axis I diagnoses (*n*=184).

*Note*. SCID = Structured Clinical Interview for DSM-IV Axis I Disorders. Axis I disorders that do not appear in this table were not diagnosed in any of the subsample. Five participants who met criteria for an Axis I disorder but not an anxiety, depressive, or adjustment disorder are not reported in this table and were not included in the analyses.

^a^No SCID diagnosis of any Axis I disorder.

^b^SCID diagnosis of one or more anxiety disorders *but no depressive disorder*. Included panic disorder with/out agoraphobia, agoraphobia without panic, specific phobia, social phobia, obsessive-compulsive disorder, post-traumatic stress disorder, generalized anxiety disorder, anxiety disorder not otherwise specified, and for the purposes of this analysis, an adjustment disorder characterized by anxiety.

^c^SCID diagnosis of a depressive disorder *but no anxiety disorder*. Included major depressive disorder, depressive disorder not otherwise specified, and for the purposes of this analysis, an adjustment disorder characterized by depressed mood.

^d^SCID diagnosis of *both* one or more anxiety disorders and a depressive disorder. For the purposes of this analysis, this included an adjustment disorder characterized by mixed anxiety and depressed mood.

| SCID diagnostic group | HADS score | | | | | | | | | | |
| --- | --- | --- | --- | --- | --- | --- | --- | --- | --- | --- | --- |
|  | Total | | |  | Anxiety subscale | | |  | Depression subscale | | |
|  | M | SD | Range |  | M | SD | Range |  | M | SD | Range |
| NO DX  (*n*=108) | 6.05 | 4.93 | 0–20 |  | 3.04 | 2.77 | 0–11 |  | 3.01 | 2.85 | 0–13 |
| ANX ONLY  (*n*=19) | 12.89 | 6.00 | 4–22 |  | 6.63 | 3.29 | 2–13 |  | 6.26 | 4.16 | 0–12 |
| DEP ONLY  (*n*=23) | 9.96 | 5.98 | 0–28 |  | 5.00 | 3.00 | 0–10 |  | 4.96 | 4.20 | 0–18 |
| COMORBID  (*n*=34) | 22.82 | 7.07 | 4–35 |  | 11.53 | 4.10 | 3–19 |  | 11.29 | 4.35 | 1–19 |

**Table S3.** HADS descriptive statistics for each SCID diagnostic group (*n*=184).

*Note*. HADS = Hospital Anxiety and Depression Scale. SCID = Structured Clinical Interview for DSM-IV Axis I Disorders. NO DX = no SCID diagnosis of any Axis I disorder. ANX ONLY = SCID diagnosis of one or more anxiety disorders *but no depressive disorder*. DEP ONLY = SCID diagnosis of a depressive disorder *but no anxiety disorder*. COMORBID = SCID diagnoses of *both* one or more anxiety disorders and a depressive disorder.

| HADS score | Predictor | *df* | SS | MS | *F* | *p* |
| --- | --- | --- | --- | --- | --- | --- |
| Total | SCID group | 3 | 7417 | 2472.3 | 78.29 | <.001 |
|  | Error | 180 | 5684 | 31.6 |  |  |
| Anxiety subscale | SCID group | 3 | 1907 | 635.7 | 64.69 | <.001 |
|  | Error | 180 | 1769 | 9.8 |  |  |
| Depression subscale | SCID group | 3 | 1803 | 600.9 | 49.24 | <.001 |
|  | Error | 180 | 2197 | 12.2 |  |  |

**Table S4.** ANOVA test statistics: HADS scores between SCID diagnostic groups (*n*=184).

*Note*. ANOVA = analysis of variance; HADS = Hospital Anxiety and Depression Scale. SCID = Structured Clinical Interview for DSM-IV Axis I Disorders. *df* = degrees of freedom. SS = sum of squares. MS = mean square.

|  | Comparison | | |  |  |  |  |
| --- | --- | --- | --- | --- | --- | --- | --- |
| HADS score | SCID group |  | SCID group | Raw mean difference | 95% familywise CI^a^ | Adjusted *p*^a^ | Cohen’s *d* |
| Total | NO DX | – | ANX ONLY | -6.85 | [-10.47, -3.22] | <.001 | 1.25 |
|  | NO DX | – | DEP ONLY | -3.91 | [-7.26, -0.56] | .01 | 0.71 |
|  | NO DX | – | COMORBID | -16.78 | [-19.64, -13.91] | <.001 | 2.75 |
|  | ANX ONLY | – | DEP ONLY | 2.94 | [-1.58, 7.46] | .33 | 0.49 |
|  | ANX ONLY | – | COMORBID | -9.93 | [-14.10, -5.75] | <.001 | 1.51 |
|  | DEP ONLY | – | COMORBID | -12.87 | [-16.80, -8.93] | <.001 | 1.96 |
| Anxiety subscale | NO DX | – | ANX ONLY | -3.59 | [-5.62, -1.57] | <.001 | 1.18 |
|  | NO DX | – | DEP ONLY | -1.96 | [-3.83, -0.10] | .04 | 0.68 |
|  | NO DX | – | COMORBID | -8.49 | [-10.09, -6.89] | <.001 | 2.43 |
|  | ANX ONLY | – | DEP ONLY | 1.63 | [-0.89, 4.15] | .34 | 0.52 |
|  | ANX ONLY | – | COMORBID | -4.90 | [-7.23, -2.57] | <.001 | 1.32 |
|  | DEP ONLY | – | COMORBID | -6.53 | [-8.72, -4.33] | <.001 | 1.82 |
| Depression subscale | NO DX | – | ANX ONLY | -3.25 | [-5.51, -1.00] | .001 | 0.91 |
|  | NO DX | – | DEP ONLY | -1.95 | [-4.03, 0.13] | .08 | 0.54 |
|  | NO DX | – | COMORBID | -8.28 | [-10.07, -6.50] | <.001 | 2.25 |
|  | ANX ONLY | – | DEP ONLY | 1.31 | [-1.50, 4.12] | .62 | 0.31 |
|  | ANX ONLY | – | COMORBID | -5.03 | [-7.63, -2.44] | <.001 | 1.18 |
|  | DEP ONLY | – | COMORBID | -6.34 | [-8.78, -3.89] | <.001 | 1.48 |

**Table S5.** Post-hoc comparisons: HADS scores between SCID diagnostic groups.

*Note*. HADS = Hospital Anxiety and Depression Scale. SCID = Structured Clinical Interview for DSM-IV Axis I Disorders. CI = confidence interval. NO DX = no SCID diagnosis of any Axis I disorder (*n*=108). ANX ONLY = SCID diagnosis of one or more anxiety disorders *but no depressive disorder* (*n*=19). DEP ONLY = SCID diagnosis of a depressive disorder *but no anxiety disorder* (*n*=23). COMORBID = SCID diagnoses of *both* one or more anxiety disorders and a depressive disorder (*n*=34). Post-hoc comparisons were conducted using Tukey’s honest significant difference.

^a^Adjusted for multiple comparisons.
